# Aortic injury caused by esophageal foreign body-case reports of 3 patients and literature review

**DOI:** 10.1097/MD.0000000000020849

**Published:** 2020-06-26

**Authors:** Liping Zeng, Wenbo Shu, Honghai Ma, Jian Hu

**Affiliations:** Department of Thoracic Surgery, The First Affiliated Hospital, College of Medicine, Zhejiang University, Hangzhou, Zhejiang Province, China.

**Keywords:** aortoesophageal fistula, esophagus, foreign body, thoracic aortic injury

## Abstract

**Objectives:**

Ingestion of a foreign body can cause different degrees of damage to esophagus, and several complications are potentially life-threatening if not properly handled. The aortic injury caused by a perforating esophageal foreign body is rare but lethal. The optimal management still remains controversial. The purpose of this report is to describe our experience in the management of the aortic injury caused by esophageal foreign body ingestion.

**Methods:**

Between January 2015 and December 2015, we retrospectively enrolled cases of esophageal perforation involving the aorta by foreign body. The general parameters, esophageal foreign body, types of aortic injury, treatment, and outcome were analyzed. Additionally, we reviewed the literature of the management of esophageal perforation involving the aorta caused by foreign bodies. The study was approved by the ethics committee of the First Affiliated Hospital, College of Medicine, Zhejiang University, and the need for informed consent was waived (Quick review 2019, No. 609).

**Results:**

Three cases of esophageal perforation involving the aorta by foreign body was selected in the study. Two male and 1 female patients (range, 51–58 years old) with the aorta involvement caused by a perforating foreign body in the esophagus in 3 forms were identified, including 1 patient with mycotic aortic pseudoaneurysm, 1 patient with aortoesophageal fistula and 1 patient with the aortic intramural hematoma. One patient died of the rupture of the pseudoaneurysm during the preparation of the surgery. The other 2 patients were cured with a multidisciplinary approach, which is an urgent thoracic endovascular aortic repair followed by mediastinal debridement/drainage or endoscopic retrieval. Two of 3 patients were survived until now.

**Conclusion:**

The management of the aortic injury caused by esophageal foreign body injury is challenging. Early diagnosis and multidisciplinary management is crucial.

## 1 Introduction

Ingestion of a foreign body can cause different degrees of damage to the esophagus, which is related to the nature, shape, and existence time of the foreign body. Generally, these patients present to ENT or GI clinics. However, several complications are potentially life-threatening if not properly handled, including esophageal perforation, mediastinal infection/abscess, and aortoesophageal fistula (AEF). The incidence of aorta involvement is rare. However, the recorded survival is dismal.^[1,2]^ Until 1980, the first recorded survival of aorta-esophageal fistula induced by a foreign body was reported by Ctercteko.^[1]^ The approach for the aortic injury following foreign body perforation of the esophagus is various. However, the techniques reported were generally applied in exceptional single cases. The optimal management still remains controversial. To our knowledge, no widely accepted guideline is available currently. On the basis of the studies reported, the role of multidisciplinary treatment is emphasized in the management of this disorder.

In this study, we retrospectively enrolled cases of esophageal perforation involving the aorta by foreign body in 2015, and in-hospital events and follow-up were evaluated (Table 1). Additionally, we reviewed the literature of the management of esophageal perforation involving the aorta caused by foreign bodies. This retrospective study was reviewed by the Institutional Review Board of our hospital. The study was approved by the ethics committee of the First Affiliated Hospital, College of Medicine, Zhejiang University (convenient review, No. 609/2019). This study conforms to ethic standard of Declaration of Helsinki.

**Table 1:**
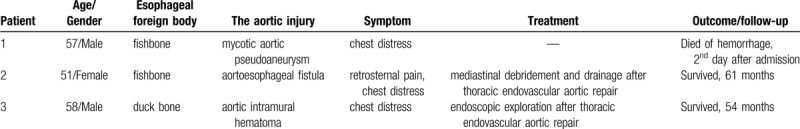
Patients’ characteristics and outcome.

## 2 Case 1 description

**Figure 1:**
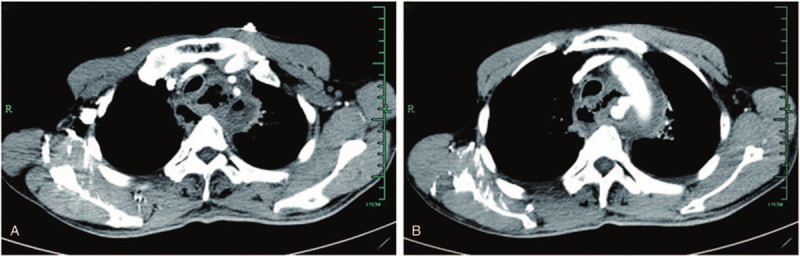
Representative images of case 1. (a) a swollen thoracic esophagus with several mediastinitis, pneumomediastinum on contrast-enhanced chest CT. (b) a pseudoaneurysm located in the aortic arch on ontrast-enhanced chest CT.

A 57-year-old man was admitted to our emergency room (ER) on Jan 07, 2015. The patient developed recurrent chest distress for 7 days after the ingestion of a fishbone. The vital signs were stable on arrival at the hospital. Laboratory findings showed a white blood cell (WBC) count of 17.1 × 109/L with 82.4% of neutrophil percentage (NP), c-reactive protein (CRP) 185.10 mg/L, procalcitonin (PCT) 2.38 ng/ml. Contrast-enhanced chest CT was performed and revealed a swollen thoracic esophagus with several mediastinitis, pneumomediastinum (Fig. 1a) and an pseudoaneurysm located in the aortic arch (Fig. 1b). Esophageal perforation caused by fishbone resulting in mediastinitis and mycotic pseudoaneurysm of the thoracic aorta was considered. We applied a treatment with broad-spectrum antibiotics, meanwhile, a curative plan was made after a multidisciplinary consultation, which was mediastinal debridement and drainage by general thoracic surgeons after thoracic endovascular aortic repair by vascular surgeons. The patient died during the preoperative preparation for stent implantation. After a sudden cough, the patients complained several chest distress and dysphoria. Anti-shock treatment was carried out immediately. However, the vital signs still became unstable and the patients died in several minutes. A bedside sonography examination was performed and showed a severe left hyperechoic pleural effusion. The sudden rupture of the pseudoaneurysm was considered as the cause of death. The patient died at the 2nd day after admission.

## 3 Case 2 description

**Figure 2:**
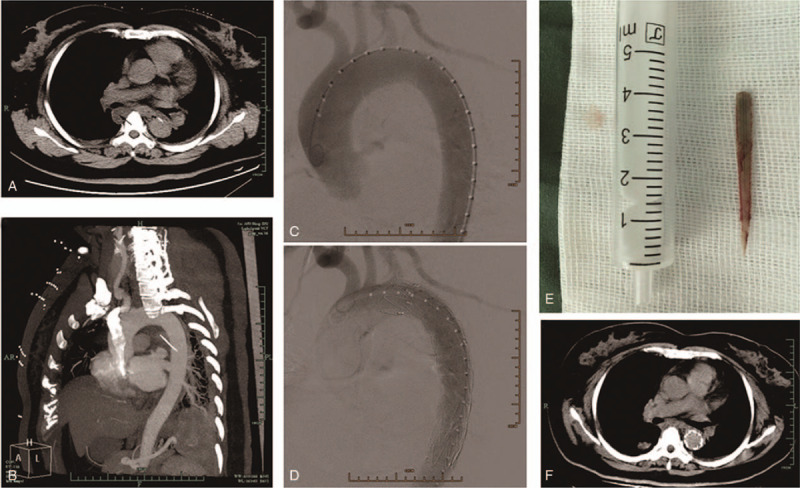
Representative images of case 2. (a) Chest CT at admission revealed a foreign body perforating the thoracic esophagus and aorta. Besides, an inferior right mediastinal cyst (arrow) was noted. (b) A subsequent CTA demonstrated a linear hyperdensity foreign body puncturing into the descending aorta, with no sigh of leakage of contrast agents and formation of hematoma. (c) The angiographic catheter was guided into the thoracic aorta and arteriography revealed no sign of pseudoaneurysm and leakage of contrast agent. (d) An endograft (28 × 150 mm, Medtronic, Inc., USA) was placed inside the descending aorta. (e) The removed fishbone during the surgery. Then, mediastinal debridement and copious irrigation was performed. (f) Chest CT scan at 1 month follow-up confirmed successful closure of the fistula.

A 51-year-old female was admitted to our ER on January 25, 2015. The patient was presented with laryngeal pain 2 days ago after consuming fish. Then, the pain was transferred to retrosternal area, which was persistent, unbearable, accompanied with chest congestion. The physical examination result was insignificant. Laboratory analysis reported a WBC count of 12.9 × 109/L with 89.7% of NP. Chest CT at admission revealed a foreign body perforating the thoracic esophagus and the thoracic aorta (Fig. 2a). Besides, an inferior right mediastinal cyst (arrow) was noted (Fig. 2a). A subsequent CTA (Fig. 2b) demonstrated a linear hyperdensity foreign body puncturing into the descending aorta, with no sign of leakage of contrast agents and formation of hematoma. After an emergent multidisciplinary consultation, an urgent thoracic endovascular aortic repair was scheduled, followed by mediastinal debridement and drainage.

Thoracic endovascular aortic repair was performed under general anesthesia. The endovascular device was inserted through the right femoral artery. The angiographic catheter was guided into the thoracic aorta and arteriography revealed no sign of pseudoaneurysm and leakage of contrast agent (Fig. 2c). Then, an endograft (28 × 150 mm, Medtronic, Inc., USA) was placed inside the descending aorta (Fig. 2d). Intraoperative angiography confirmed successful results, and the patient was transferred for further thoracic exploration. Right anterior-lateral min-thoracotomy through 5th intercostals space was performed. The mediastinal mass (3 × 2 cm) was resected firstly. The suspected injured part of esophagus was dissected. Mild inflammatory exudates were observed and a fishbone penetrating the esophagus and puncturing into the thoracic aorta was noted. The fishbone (Fig. 2e) was removed gently. Then, mediastinal debridement and copious irrigation was performed. A silastic flexible tube connecting to a negative pressure drainage ball was placed in the esophageal bed for mediastinal drainage. Besides, another 2 chest tubes were placed for chest drainage and irrigation with 0.5% (v/v) povidone iodine. Supportive treatments-including nihil per os (NPO) status, intravenous broad-spectrum antibiotics, nasogastric tube decompression, total parenteral nutrition (TPN), and proton pump inhibitors (PPIs), were administered after surgery. A gastrojejunal tube for early enteral nutrition support was placed by endoscopy on the third postoperative day. The oral methylene blue test was negative and the patients started oral intake on the 14th postoperative day. Subsequently, all the drainage tubes were removed and the patient was discharged on the 16th postoperative day on broad-spectrum oral antibiotics. One month after discharge, the follow-up Chest CT scan confirmed successful closure of the fistula (Fig. 2f). The patient was asymptomatic and alive at 61-month follow-up.

## 4 Case 3 description

**Figure 3:**
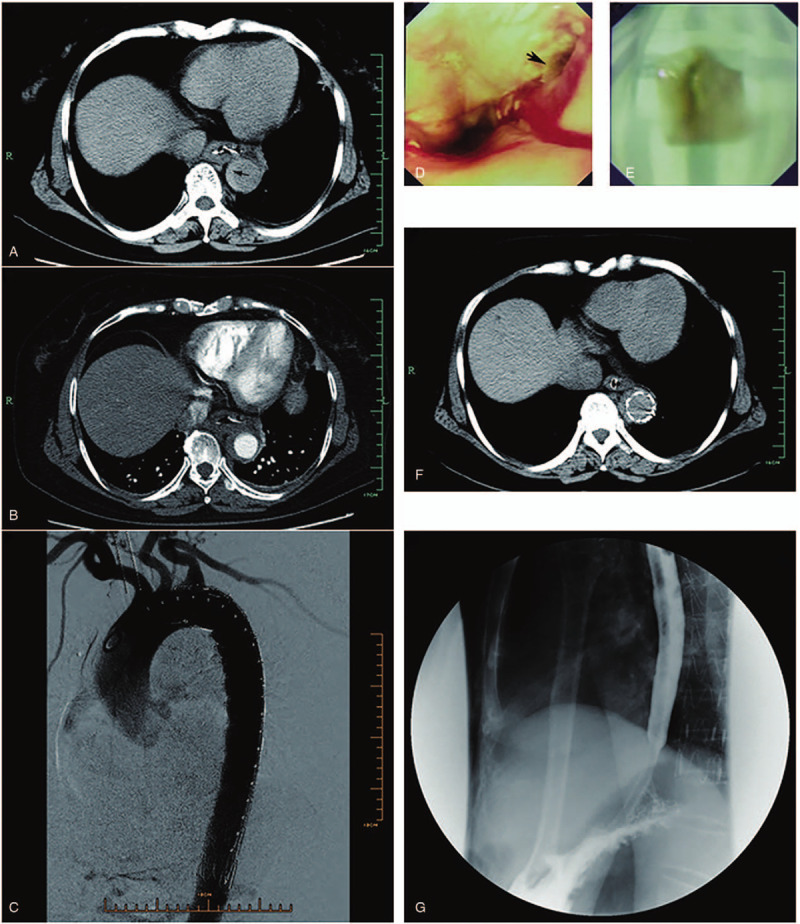
Representative images of case 3. (a) Chest CT was performed and revealed a lower esophageal foreign body and crescent wall thickening in the descending aorta (arrow). (b) Subsequent CTA demonstrated a descending IMH. (c) An endograft (34 × 200 mm, Lifetech Scientific, China) was successfully placed inside the descending aorta. Postoperative angiography confirmed successful results. (d) Endoscopy showed a foreign body (arrow) in the lower third of the esophagus, stabbing into the esophageal wall. Esophageal mucosal injury and fresh bleeding was also observed. (e) The removed duck bone (2 × 3 cm). (f) The chest CT (August 26, 2015) revealed no sign of mediastinal infection. (g) Esophagography (August 27, 2015) with diatrizoate meglumine test was negative.

A 58-year-old man was admitted to our ER on August 15, 2015. The patient suffered from chest distress for 4 days after ingestion of a duck bone. Chest X-ray at the local hospital revealed a lower esophageal foreign body. The outpatient endoscopic treatment was failed. He was transferred to our hospital for further treatment. Chest CT (Fig. 3a) was performed and revealed a lower esophageal foreign body and crescent wall thickening in the descending aorta (arrow). Subsequent CTA confirmed a descending intramural hematoma (Fig. 3b). After a multidisciplinary consultation, including vascular surgeon, GI physician, and general thoracic surgeon, a combined treatment approach was planned.

Thoracic endovascular aortic repair was performed under general anesthesia. The endovascular device was inserted through the right femoral artery. The angiographic catheter was guided into the thoracic aorta and arteriography revealed no sign of dissection and leakage of contrast agent. Then, an endograft (34 × 200 mm, Lifetech Scientific, China) was placed inside the descending aorta (Fig. 3c). Postoperative angiography confirmed successful results, then, the endoscopic exploration was performed. Endoscopy (Fig. 3d) showed a foreign body (arrow) in the lower third of the esophagus, stabbing into the esophageal wall. Esophageal mucosal injury and fresh bleeding was also observed. The duck bone (2 × 3 cm, Fig. 3e) was removed gently. After endoscopic hemostasis of norepinephrine solution irrigation, the endoscope was removed. Supportive treatments-including NPO status, intravenous broad-spectrum antibiotics, nasogastric tube decompression, TPN and PPI were given. The chest CT (Fig. 3f, August 26, 2015) revealed no sign of mediastinal infection and esophagography (Fig. 3g, August 27, 2015) with diatrizoate meglumine test was negative. The patients started liquid intake on the 13th postoperative day (August 28, 2015). Subsequently, the patient was discharged on the 16th postoperative day. The patient was asymptomatic and alive at 54-month follow-up.

## 5 Discussion

Aortic injury following foreign body perforation of the esophagus is rare. In this study, 3 types of aortic injury were illustrated, including mycotic pseudoaneurysm, AEF and aortic intramural hematoma.

Classically, AEF is described as the communication between the aorta and esophagus, which is mainly caused by thoracic aortic disease and esophageal disease. Esophageal foreign body complicated with AEF is mostly reported in cases, caused by foreign body directly stab or infection invading the main vein wall, causing the rupture. Its typical manifestation is high fever, retrosternal pain, and hematemesis. According to a large series study of 2394 patients with a foreign body in the esophagus treated between 1965 and 1976, 2 patients in the series developed esophagoaortic fistula.^[3]^ Zhang X et al reported that among 3209 cases of esophageal foreign body in their center between 1963 and 2010,^[2]^ 32 cases (1%) were complicated with aortoesophageal fistula but only 3 patients (9%, 3/32) survived. Canaud et al conducted a systematic review of thoracic endovascular aortic repair (TEVAR) for AEF.^[4]^ Their study included 72 patients treated by TEVAR for AEFs. After a mean follow-up of 7.4 months (range, 1–33 months), the all-cause mortality was high as 40.2%, and the aortic-related mortality was 33.3%. Prolonged antibiotic treatment (*P* = .001) and repair of AEFs due to a foreign body. AEF is associated with thoracic aortic aneurysm, esophageal cancer, previous thoracic aortic surgery, foreign bodes, etc.^[5–10]^ Although the first recorded survival of AEF induced by a foreign body was reported by Ctercteko in 1980,^[1]^ the morbidity rate of patients with AEF remain extremely high, and the management of this life-threatening condition is unclear. To date, several types of treatment have been described, such as surgery, endovascular intervention, combined repair, etc.

Surgical repair is the typical treatment approach and considered as the only definitive treatment for foreign body-related AEF. According to a study including 32 cases of esophageal foreign body complicated with aortoesophageal fistula,^[2]^ all 13 non-surgically treated cases died while 3 of 19 surgically treated patients were completely cured. A left thoracotomy followed by aortic replacement with a prosthetic/cryo preserved homograft is a typical approach for open AEF repair.^[8,11]^ In some centers, the choice of in situ aortic allograft replacement is preferred.^[12]^ Sometimes, an extra-anatomic bypass would be needed.^[8]^ Importantly, thorough debridement of all contaminated tissues and copious irrigation of the thorax is essential. However, open surgery repair is technically demanding, accompanied with high mortality rates.

Compared with traditional open surgery for the treatment of aorta fistulas, Endovascular stent repair for AEF has been described with good results.^[13,14]^ However, for patients with AEF, endovascular treatment alone is highly risky because of infectious complication, which could result in delayed death.^[13–15]^ In addition to the already presence of infection, the perforation of the esophagus is not repaired and the contamination would be deteriorated. According to a previous case reported,^[16]^ a patient with aortoesophageal fistula caused by a descending aortic pseudoaneurysm recovered after endoluminal stent repair and intravenous antibiotic treatment. However, the patient was re-admitted 3 weeks later because of severe fever. Eventually, the patient died of an exsanguinating hemorrhage, and the infected aortic stent graft was considered as the cause. Another study reported that the endovascular management of 2 cases of aortoenteric and aortoesophageal fistula that were unsuccessfully treated with an aortic stent graft because of recurrent infection.^[15]^ In general, it should be considered as a bridge procedure for further treatment in the acute situation, preventing fatal deteriorate on. Additional open surgical repair was performed in several cases and sometimes no additional therapy was offered.^[10,17]^ However, Patient's with AEFs and clinical signs of infection who are in critical physical condition that makes them at high risk for open surgery should be considered for endovascular surgery as a palliative treatment, or a temporary alternative until they are healthy enough to tolerate open surgery. There have been cases reported of the successful use of endografts as a provisional treatment in patients with aortoduodenal and ilioenteric fistulas that allowed improvement of their general condition until definitive treatment by open surgery was possible. Although endovascular repair appears to be a promising therapeutic modality, in the presence of infection this technique should be considered on an individual basis.^[18]^ Failure of treatment should be expected in a significant number of cases during follow-up, particularly in patients with signs of sepsis. In some cases, esophagoscopy may induce sudden death from massive hemorrhage if AEF is already present.

Pseudoaneurysms, differ from true aneurysms in that they lack all 3 natural layers of arterial wall, resulting in extravasation of arterial blood outside of the artery. The main etiology is trauma, inflammation, and iatrogenic causes.^[19–21]^ The high-pressure blood in the artery squeezes into the tissue around the damaged artery and forms a perfusion sac that communicates with arterial lumen, which is contained by the media or adventitia, or only by the soft-tissue structures surrounding the injured vessel.^[22]^ Mycotic aortic pseudoaneurysm is an uncommon disease associated with a high mortality rate, mainly caused by direct or indirect infection of aorta by pathogenic microorganisms. Aortic pseudoaneurysms caused by esophageal foreign bodies are generally considered to be esophageal foreign bodies causing perforation of the esophagus, which leads to the secondary mediastinal infection. The mediastinal infection invades the aortic wall, producing infectious aneurysms. The gold standard of therapy is open repair of mycotic descending thoracic aortic aneurysms, including aggressive intraoperative debridement with in situ prosthetic reconstruction and Lifelong antibiotic suppression therapy.^[23]^ Traditional open surgery requires high technical conditions, but the treatment effect is limited, and the perioperative mortality rate is high.^[24]^ In recent years, with the development of materials science, the use of endovascular repair combined with antibacterial drugs has also become one of the treatment methods, which can reduce surgical trauma and simplify operation, and is especially suitable for high-risk patients.^[25]^ Endovascular repair can control active bleeding effectively and pull the patient temporarily out of danger, but cannot remove the infection, which remains a risk of delayed massive bleeding. It is recommended that after thoroughly evaluation of each patient, we should use conventional open repair, endovascular repair, or a combination of both approaches, and treated on a case-by-case basis.^[26]^ Meanwhile, it is crucial to reinforced related follow-up to monitor recurrence of pseudoaneurysms and reduce long-term complications.

Aortic intramural hematoma (IMH) is defined as a hematoma confined within the medial layer of the aorta in the absence of a detectable intimal tear and without connection with vascular cavity, which is a special subtype of aorta dissection.^[27]^ The mechanism of IMH has been reported including the rupture of aortic middle layer nourish vessels causing the hematoma, atherosclerosis; iatrogenic injury, or trauma. There are still many controversies in IMH treatment, focusing on whether to take surgery, surgical timing, and surgical methods. For Stanford type A IMH involving the ascending aorta, patients with unstable condition should be treated as soon as possible, and patients with relatively stable disease could be treated conservatively for a period of time before surgical treatment. The treatment of Stanford type B intramural hematoma, which is confined to the descending aorta, is currently controversial. Patients with stable disease may consider conservative treatment temporarily, but strict monitoring is required.^[28]^ If subsequent hematoma progression and complications (aortic dissection, aortic ulcer, aortic aneurysm) occur, surgical treatment should be aggressively performed.^[29,30]^ In case 3, we considered it was the esophageal foreign body caused IMH, and the endoscopic examination may exacerbate the aortic injury. So, in the third case, we managed successfully by combined endovascular treatment by means of a stent graft and followed endoscopic management. The patient is currently in good condition and has achieved long-term survival.

In summary, aortic injury caused by esophageal foreign body can cause severe complications such as AEF. With appropriate attention to each of these components, a successful outcome can be achieved with multidisciplinary treatment approaches.

## Author contributions

Liping Zeng designs the study, collects the data, analyzes the cases and writes the first draft. Wenbo Shu and Honghai Ma revises the subsequent versions of the manuscript. All authors read and approved the final manuscript.
